# Development and Validation of a Multiplex, Real-Time RT PCR Assay for the Simultaneous Detection of Classical and African Swine Fever Viruses

**DOI:** 10.1371/journal.pone.0071019

**Published:** 2013-07-26

**Authors:** Felicity J. Haines, Martin A. Hofmann, Donald P. King, Trevor W. Drew, Helen R. Crooke

**Affiliations:** 1 Virology Department, Animal Health and Veterinary Laboratories Agency, New Haw, Surrey, United Kingdom; 2 Development Department, Institute of Virology and Immunoprophylaxis, Mittelhäusern, Switzerland; 3 The Pirbright Institute, Pirbright Laboratory, Pirbright, Surrey, United Kingdom; USGS National Wildlife Health Center, United States of America

## Abstract

A single-step, multiplex, real-time polymerase chain reaction (RT-PCR) was developed for the simultaneous and differential laboratory diagnosis of Classical swine fever virus (CSFV) and African swine fever virus (ASFV) alongside an exogenous internal control RNA (IC-RNA). Combining a single extraction methodology and primer and probe sets for detection of the three target nucleic acids CSFV, ASFV and IC-RNA, had no effect on the analytical sensitivity of the assay and the new triplex RT-PCR was comparable to standard PCR techniques for CSFV and ASFV diagnosis. After optimisation the assay had a detection limit of 5 CSFV genome copies and 22 ASFV genome copies. Analytical specificity of the triplex assay was validated using a panel of viruses representing 9 of the 11 CSFV subgenotypes, at least 8 of the 22 ASFV genotypes as well as non-CSFV pestiviruses. Positive and negative clinical samples from animals infected experimentally, due to field exposure or collected from the UK which is free from both swine diseases, were used to evaluate the diagnostic sensitivity and specificity for detection of both viruses. The diagnostic sensitivity was 100% for both viruses whilst diagnostic specificity estimates were 100% for CSFV detection and 97.3% for ASFV detection. The inclusion of a heterologous internal control allowed identification of false negative results, which occurred at a higher level than expected. The triplex assay described here offers a valuable new tool for the differential detection of the causative viruses of two clinically indistinguishable porcine diseases, whose geographical occurrence is increasingly overlapping.

## Introduction

Classical swine fever (CSF) and African swine fever (ASF) are two contagious viral diseases of swine affecting both domestic and wild *Suidae* populations of all breeds and ages. Both diseases are notifiable to the World Organisation for Animal Health (OIE) due to the high mortality rates associated with the acute forms of both diseases, and the potential for rapid spread and huge economic loss incurred by affected countries, along with impact for international trade.

CSF is caused by Classical swine fever virus (CSFV), an enveloped single-stranded, positive-sense RNA virus belonging to the *Pestivirus* genus of the *Flaviviridae* family [1]. CSFV is related to the other pestiviruses, Bovine viral diarrhoea virus type I (BVDV-1) and type II (BVDV-2), Border disease virus (BDV), pestivirus of giraffe and ‘HoBi-like’ atypical pestiviruses [2]. The approximately 12.3 kb CSFV genome encodes a single large open reading frame (ORF) flanked by highly conserved 5′- and 3′-untranslated regions (UTRs). CSFV has a worldwide distribution and, although the virus has been eradicated in many countries, the disease was present in 46 regions or countries between January 2005 and August 2012 [3].

ASF is caused by African swine fever virus (ASFV); a large, enveloped double-stranded DNA virus belonging to the *Asfivirus* genus of the *Asfarviridae* family [4]. ASFV infects all *Suidae* including domestic pigs, wild boar and warthogs and is currently endemic in Sardinia and most sub-Saharan countries of Africa [3]. Until recently, transcontinental spread of ASFV was rare, but in 2007, ASFV was detected in domestic pigs in Georgia and within a number of months had been confirmed in the neighbouring countries of Armenia and Azerbaijan and the Russian republic of Chechnya [5]. In 2011, ASFV had spread from the Caucasus region to south-west Russia, likely via wild boar and also to north-west and west Russia in a series of “jumps” [6]–[8] and in 2012 was reported in the Ukraine [3].

African swine fever cannot be differentiated from CSF by either clinical presentation or post mortem examination, therefore in the event of a suspected outbreak, differential diagnosis of the two diseases is essential. Control measures for containment of both CSFV and ASFV in the event of an outbreak in the EU are based on stamping out of animals in affected and suspect holdings [9], sometimes without prior virus detection (“pre-emptive culling”). CSF has recently been reported in Hungary, Lithuania, Serbia, Russia and Latvia [3], [7], [8] and, with the spread of ASFV from the Caucasus, the likelihood of countries encountering both CSF and ASF is increased. This emphasizes the need for effective differential diagnosis of either disease to prevent, or at least limit, substantial economic losses to the swine industry.

Multiplex, gel-based PCR assays for detection of CSFV and ASFV [10], [11] and RT-PCR assays for singleplex CSFV [11]–[18] or ASFV detection [19]–[21] have been described. However, to date, a multiplex real-time RT-PCR for simultaneous detection of CSFV and/or ASFV has not been reported.

The objective of this study was to develop a multiplex assay for differential diagnosis of CSFV and ASFV alongside an exogenous internal control. The incorporation of an exogenous internal control increases the reliabilty of the RT-PCR results by permitting discrimination of true negative results from false negatives which may occur due to improper nucleic acid extraction or the presence of PCR inhibitors in the sample. The assay has been validated on material from experimentally infected animals and from positive and negative field samples and provides a one-tube molecular detection system for differential diagnosis of two clinically indistinguishable diseases.

## Materials and Methods

### Ethical Statement

Experimental infections with Classical swine fever were approved by the Animal Health and Veterinary Laboratories Agency ethics committee. African swine fever positive material was derived from experimental infections approved by the ethics committees of the Pirbright Institute and the Institute of Virology and Immunoprophylaxis. All procedures conducted in the UK were in accordance with the UK Animals (Scientific Procedures) Act 1986 under project license permit numbers PPL 70_6559 (CSFV) and PPL 70_5029 (ASFV). Procedures conducted in Switzerland involving infectious ASFV were approved by the Bundesamt für Umwelt, Abteilung Abfall, Stoffe, Biotechnologie under the license number A000205-O1-4D. The experiments were authorized by the Cantonal Ethics Committee (kantonale Tierversuchskommission). To ameliorate suffering animals were observed at frequent, regular intervals and clinical score sheets completed which informed euthanasia decisions. Animals were euthanized when ever the first of either the predefined scientific or humane endpoints was reached.

### Viruses and viral nucleic acid

Panels of 9 CSFV and 30 ASFV of varying virulence, genotype, geographical origin, sample type and year of isolation were used in this study (Table 1). The selection comprised representatives of 9 of the 11 CSFV subgenotypes and 8 ASFV genotypes, including a genotype II isolate from Georgia, along with several ASFV samples of undetermined genotype. Other pestiviruses included representatives of BDV and BVDV types I and II. All pestiviruses used in this study were maintained at the Animal Health and Veterinary Laboratories Agency (AHVLA), Weybridge, UK, whilst ASFV samples were obtained from the Pirbright Institute, UK. DNA from 14 ASFV strains was provided by the Instituto Nacional de Investigacion (INIA), Madrid, Spain.

**Table 1:** Viruses used to determine analytical specificity of the triplex RT-PCR assay.

Virus	Subgenotype/genotype	Isolate
**CSFV**	1.1	Alfort 187
	1.2	Brescia
	1.3	Guatemala HC#4009
	2.1	UK2000/7.1
	2.2	SP399/96
	2.3	Rostock
	3.1	Congenital Tremor
	3.3	CBR/93
	3.4	Kanagawa
**ASFV**	I	Ang72*, Ba71V*, Ben 97/1, BF07*, CV97*, Dakar 59, E70*, E75*, Haiti*, HOLLAND 86, Lis 60, ORI 84, ORI 85, Ss88*,
	II	Grg 2007/8
	III	
	IV	
	V	Moz 64*, Moz 79, TEN 89/1
	VI	
	VIII	Lil 90/1, MwLil20/1*, DOWA, MAN 89/2
	IX	Ken06.B1*, Ken07.Eld1*, Uga 95/1, Ug03H*
	X	Bur 84/1, BUR 90/2
	XVII	ZIM 92/1
	XXI	RSA/1/96
	Not determined	CAM 1/87 P4, CAM 89/7, CON 2006/1, DEIGHTON, KAN B 89/2, KEF 89/2, KEF 89/9, MALTA MSF2A, MGR 1/2005, MVMT 90/1, NUR 86, SIY 92/1, UGA 95/2
**BVDV I**	1a	C24V
**BVDV II**	2a	502643
**BDV**	1	S137/4

* Samples obtained from Instituto Nacional de Investigacion in Madrid, Spain.

All other ASFV samples were obtained from The Pirbright Institute, UK.

All CSFV strains were obtained from AHVLA reference collection.

### Clinical samples from negative and experimentally infected animals

A total of 45 EDTA blood and 54 pooled tissue homogenate samples were collected from 49 animals experimentally infected with CSFV strain UK2000 7.1 or CBR/93 [22]–[24]. Tissue pools from individual animals consisted of tonsil and mandibular lymph node; kidney and spleen or mesenteric lymph node and ileum. These pooled tissue samples were homogenized by grinding approximately 0.5 gram of tissue with sand in Griffiths tubes in 4.5 ml PBS [14]. Blood samples were obtained from experimentally infected pigs at various time points after inoculation or at post-mortem. A total of 6 EDTA blood and 30 tissue homogenates (spleen, kidney, tonsil, mandibular lymph node or mesenteric lymph node) from 6 animals experimentally infected with ASFV strain Lil90, were also used. An additional panel of negative control material comprising 80 EDTA blood and 18 pooled tissue homogenates (as above) were collected from 86 pigs post euthanasia.

### Field samples

A selection of 31 archived EDTA blood samples from pigs found to be CSFV-positive during the UK CSFV outbreak in 2000 [25] and 30 ASFV-positive tissue homogenates, serums and EDTA blood samples from archived field samples submitted to The Pirbright Institute for detection of ASFV, were analysed.

### Artificial templates

Artificial templates were used for quantification of viral copy numbers and analysis of analytical sensitivity. A plasmid, pCRXLV324-6, containing the 5′-UTR of CSFV strain Alfort 187 [23] was used to generate *in vitro* RNA transcripts using the Megashortscript kit (Ambion). A plasmid, pASFV-VP72, containing a fragment of the ASFV VP72 gene [19] was linearised with *Sal* I. Nucleic acid of both artificial templates was quantified using a NanoDrop (Thermo Scientific) spectrophotometer (OD_260nm_), and stored at −20°C. Ten-fold dilution series were prepared for each nucleic acid template. An *in vitro* transcript of the enhanced green fluorescent protein (EGFP) gene (QIAGEN) was used as an internal control RNA (IC-RNA) at the concentration recommended by the manufacturer (8×10^4^ copies IC-RNA per µl).

### Nucleic acid isolation

Nucleic acid (DNA and RNA) was isolated from samples using the QIAamp mini viral RNA kit (QIAGEN) which, as detailed in the kit's handbook, is also suitable for the extraction of DNA and has been used successfully for ASFV DNA isolation [19]. Manufacturer's instructions were modified by the addition to the lysis buffer of one tenth of the final elution volume of internal control RNA (IC-RNA), equating to 8×10^4^ copies IC-RNA per µl of nucleic acid eluate (QIAGEN). Briefly, 140 µl of sample were added to 560 µl AVL lysis buffer with 6.5 µl IC-RNA, vortexed and incubated at room temperature for 10 min. Nucleic acids were then extracted according to the manufacturer's instructions, eluted in 65 µl elution buffer and stored at −20°C.

### Primers and probes

Primers and probes used in this study are listed in Table 2. Primers specific for the 5′-UTR of CSFV, CSF 100-F and CSF 192-R and TaqMan probe, CSF-Probe 1, which was modified with a BHQ-1 quencher, were used alongside IC-RNA-specific primers EGFP1-F and EGFP2-R and TaqMan probe, EGFP1-HEX [12], [26]. A TaqMan probe, termed ASFV-Cy5, designed to the 3′ end of the ASFV VP72 gene [19] was modified with a Cyan 5 dye and Black Hole Quencher II (Eurogentec, Belgium). All other oligonucleotides used were synthesized by Sigma Aldrich, UK. PCR primers specific for ASFV template detection (ASFVp72IVI_L and ASFVp72IVI_R) are described in section 3.1 and had been designed in a previous study (Hofmann, unpublished results).

**Table 2:** Primer and probes used in the triplex RT-PCR.

Target	Primer/Probe	Sequence 5′–3′
**CSFV**	CSF100-F	ATG CCC AYA GTA GGA CTA GCA
	CSF-Probe 1	*FAM*-TGG CGA GCT CCC TGG GTG GTC TAA GT-*BHQ1*
	CSF192-R	CTA CTG ACG ACT GTC CTG TAC
**ASFV**	ASFVp72IVI_L	GAT GAT GAT TAC CTT YGC TTT GAA
	ASFV-CY5	*Cy5*-CCA CGG GAG GAA TAC CAA CCC AGT G-*DDQ II*
	ASFVp72IVI_R	TCT CTT GCT CTR GAT ACR TTA ATA TGA
**IC**	EGFP1-F	GAC CAC TAC CAG CAG AAC AC
	EGFP1-HEX	*HEX*-AGC ACC CAG TCC GCC CTG AGC A-*BHQ1*
	EGFP2-R	GAA CTC CAG CAG GAC CAT G

### Optimisation of RT-PCR assays

RT-PCR optimisation was performed according to the guidelines described by Agilent Technologies [27] on artificial nucleic acid templates for CSFV or ASFV detection alongside an exogenous internal control RNA for singleplex (CSFV or ASFV), duplex (CSFV plus IC-RNA or ASFV plus IC-RNA) and triplex (CSFV, ASFV and IC-RNA) detection using the One Step SuperScript III Platinum RT-PCR kit (Invitrogen, UK).

Concentrations of primers, probes and Mg^2+^ were optimised in a final reaction volume of 25 µl to obtain maximum dRn and minimal threshold cycle (C_T_) The reaction mix contained 8.4 µl nucleic acid extract, 12.5 µl 2× reaction mix, a final concentration of 5 mM MgSO_4_, 5 U RNAsin, 50 nM ROX and 0.5 U Superscript III reverse transcriptase/Platinum Taq mix. Primers CSF100-F and CSF 192-R were used at a concentration of 600 nM, with the CSF-Probe 1 used at 200 nM whilst EGFP1-F and EGFP2-R primers were used at 200 nM and EGFP-HEX probe at 100 nM. Primers ASFVp72IVI_L and ASFVp72IVI_R were employed at a concentration of 900 nM and the ASFV-Cy5 used at 200 nM.

Samples were amplified using a Mx3005P QPCR System (Agilent Technologies) using the following conditions: reverse transcription at 50°C for 15 min, followed by incubation at 95°C for 2 min, then 50 cycles of denaturation at 95°C for 15 s and annealing and extension at 60°C for 1 min. After amplification, a threshold cycle (C_T_) value was assigned to each sample.

### Analytical and diagnostic sensitivity and specificity

A log-10 dilution series of both CSFV RNA and ASFV DNA artificial templates were produced in nuclease-free water (Promega, UK) containing IC-RNA (8×10^4^ copies per µl). Serial dilutions were then assayed in triplicate using the optimised singleplex, duplex and triplex RT-PCRs to determine analytical sensitivity of the assay and C_T_ values compared. Each test was performed on three occasions to verify repeatability and regression analysis and one-way ANOVA performed on the C_T_ values (GraphPad Prism 6). The performance of the triplex RT-PCR, was assessed using RNA extracted from cell culture samples of the panel of 9 CSFV isolates and 3 other pestiviruses. A panel of 44 ASFV DNA samples were also analysed (Table 1).

The analytical and diagnostic sensitivity and specificity of detection for each viral template using the triplex RT-PCR was compared to reference method methods, i.e. singleplex PCRs for CSFV or ASFV detection.

For CSFV detection, a PCR termed RT-nPCR-TaqMan, which is based on a method detailed in McGoldrick et al. (1999) and [28] was used as reference method. This method has been further modified with the addition of 3 µl template, amplification using a Mx3005P QPCR system and cycling conditions changed as follows: 42°C for 30 min, 94°C for 2 min and then 10 cycles at 94°C for 30 s, 60° for 30 s and 72°C for 30 s in the first step and then a second nested (nPCR) step of 94°C for 2 min followed by 40 cycles of 94°C for 30 s, 60°C for 30 s and 72°C for 30 s. Final C_T_ values derived for the RT-nPCR-TaqMan assay included an additional 10 C_T_s to account for the first step of this nested PCR.

For ASFV, a modified version of the QPCR as detailed in [19] was used as reference method. This method, termed ASFV-PCR, uses probe and primers at 250 nM and 400 nM concentrations, respectively, with a Quantifast probe PCR kit with no ROX (QIAGEN). Viral nucleic acid samples (2 µl) were added to 18 µl mastermix and the following cycling parameters used: 95°C for 3 min (Taq activation), followed by 45 cycles at 95°C for 10 s and 58°C for 60 s.

Diagnostic sensitivity for the identification of each virus was calculated as the number of true positive results, i.e. those identified as positive by both the standard PCR method and the triplex RT-PCR, divided by the sum of number of true positives and false negative results [29].

Diagnostic specificity for the identification of each virus was calculated as the number of true negative results, i.e. those identified as negative using standard PCR method and triplex RT-PCR, divided by the sum of number of true negatives and false positive results [29].

## Results

### Selection of primer, probes and optimised parameters for simultaneous detection of ASFV, CSFV and an internal control

Different combinations of a variety of primers and probes that detect ASFV or CSFV, including those described by Hoffmann et al. (2005), King et al. (2003) and McGoldrick et al. (1999) and novel primers that target the VP72 gene of ASFV, were evaluated using a selection of RT-PCR kits. New ASFV primers that bind to well conserved sequences immediately flanking the probe designed by King et al. (2003) were selected. These primers result in a shorter PCR amplicon of 75 bp, compared to 250 bp as described by King et al. (2003), and consequently remove the requirement of a separate extension step in the thermal profile. The performance of the original and the new primers was compared by testing dilution series of cell culture supernatant from ASFV-infected cells alongside serum and spleen homogenates from an ASFV-infected pig. Steeper amplification curves with lower C_T_ values and better amplification efficiency (King et al. (2003) primers: 83%, new primers: 89%) were obtained for both ASFV cell culture supernatants and tissue homogenates.

The most promising results for the detection of all three targets were obtained using the combination of primers in listed in Table 2. The RT-PCR parameters were then optimised with this primer combination.

### Analytical Sensitivity

**Figure 1:**
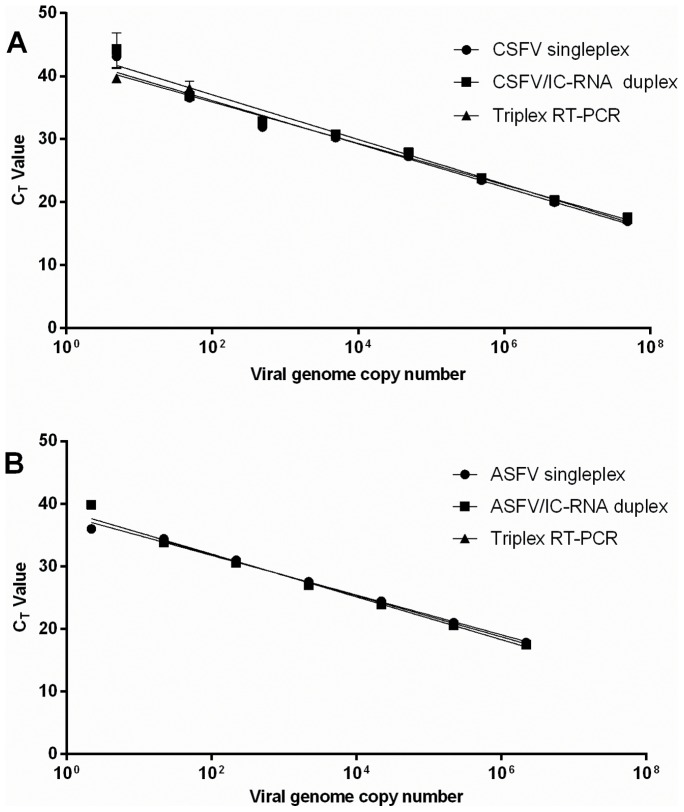
Analytical sensitivity of RT-PCR for singleplex, duplex and triplex assays for CSFV and ASFV detection. Serial dilutions of A) *in vitro* transcribed CSFV RNA or B) linearised ASFV plasmid DNA were amplified in RT-PCRs containing primers and probes to detect either single, duplex or triplex targets.

The analytical sensitivity and linearity of the optimised triplex RT-PCR was determined and compared to duplex and singleplex assays using serial dilutions of either the artificial CSFV RNA or ASFV DNA templates (Figure 1).

Standard curves (C_T_ versus log_10_ DNA or RNA copies) demonstrated linear amplification for both CSFV RNA and ASFV DNA and detection limits of 5 and 22 viral genome copies, respectively, in singleplex, duplex and multiplex reactions. Addition of IC-RNA had no effect on amplification efficiencies or correlation co-efficients. There was no significant difference between C_T_ values obtained with the singleplex, duplex and triplex assays for detection of both CSFV and ASFV targets. Regression analysis of standard curves confirmed linearity of the triplex RT-PCR (correlation co-efficient, R^2^  = 0.985; efficiency of amplification, E = 101.5%) with a dynamic range between 5 and ≥5×10^11^ copies for CSFV artificial template and between 22 and ≥2.2×10^8^ copies for ASFV artificial template (correlation co-efficient, R^2^  = 0.9991; efficiency of amplification, E = 101.5%). Internal control RNA yielded C_T_ values between 28 and 38. It was noted that in samples with high viral copy numbers, the C_T_ value for IC-RNA was absent or higher than for those samples with low viral copy numbers, suggesting a competitive inhibition for amplification of the IC-RNA.

Based on these results, the following criteria were applied for assessing unknown test samples. A CSFV sample was considered positive if the CSFV-probe (FAM) C_T_ value ≤38, whilst samples were considered ASFV positive if the ASFV-probe resulted in a (CY5) C_T_ value ≤35. Samples were classed as CSFV- and ASFV-negative if the IC-RNA-probe (HEX) gave C_T_ values ≤38 but for which no C_T_ value for FAM or CY5 were obtained. In the event where a sample did not meet the above criteria, the sample was classified as “inconclusive”.

**Figure 2:**
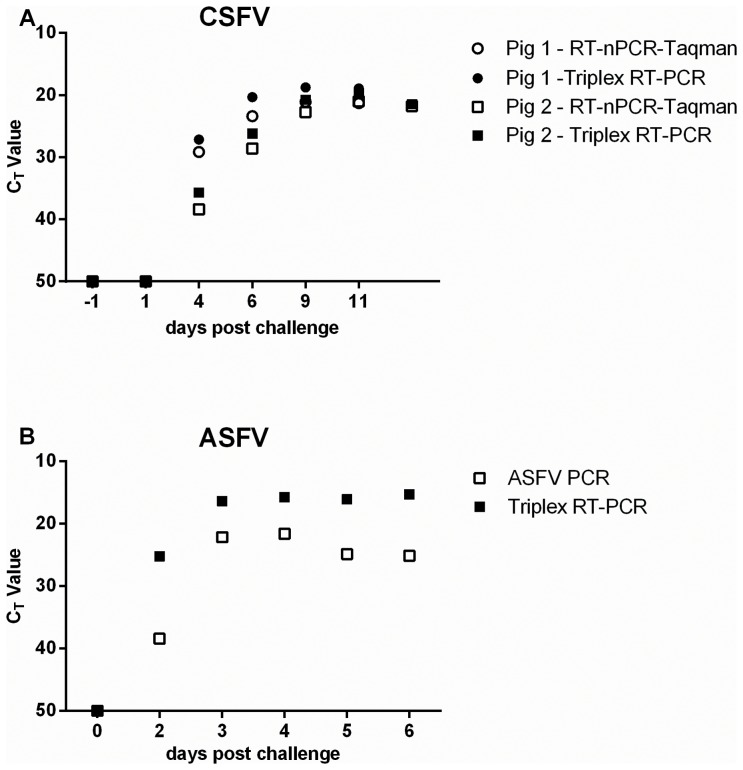
Analytical sensitivity of triplex and reference method PCRs on samples from experimentally infected animals. (A) Viral RNA, extracted from blood samples taken at various time points post challenge from 2 animals (circles or squares) experimentally infected with CSFV and analysed by triplex RT-PCR (filled symbols) or CSFV RT-nPCR-TaqMan assay (open symbols). (B) Viral RNA, extracted from homogenised spleen samples taken from 6 pigs euthanized at various time points post challenge with ASFV and analysed by triplex RT-PCR (filled symbols) and ASFV-PCR (open symbols).

To determine the sensitivity of CSFV detection in EDTA blood, samples taken from two pigs over the course of an experimental infection with CSFV UK2000/7.1 were analysed. Nucleic acid was extracted and tested with both the triplex RT-PCR and the CSFV RT-nPCR-TaqMan assay. Both methods were comparable and detected samples as CSFV positive on the same day post-infection (Fig. 2, A), demonstrating comparability of the triplex RT-PCR to the current diagnostic RT-nPCR-TaqMan method.

Detection of ASFV in clinical samples was determined using nucleic acid extracted from spleen homogenates from 6 pigs euthanized at different times post experimental infection with ASFV Lil90. Both methods detected samples to be ASFV-positive two days after infection (Figure 2, B) demonstrating comparability of the triplex RT-PCR to the reference method ASFV-PCR, with the triplex RT-PCR resulting in lower C_T_ values than the ASFV-PCR.

### Analytical Specificity

Analytical specificity distinguishes between the target analyte and other components in the assay in 3 ways, termed selectivity, exclusivity and inclusivity [30]. Nucleic acid from 12 different pestivirus strains, representing 9 of the 11 CSFV subgenotypes, 30 ASFV isolates of various genotype and a panel of 14 ASFV DNA samples were tested to determine the inclusivity of the triplex RT-PCR (Table 1). Three non-CSFV pestiviruses were tested to confirm the exclusivity of the assay. The triplex RT-PCR specifically detected RNA from all 9 CSFV isolates and DNA from the 44 diverse ASFV. There was no non-specific signal from the CSFV probe in reactions with ASFV DNA, nor for the ASFV probe with CSFV RNA. The 3 non-CSFV pestiviruses analysed were not detected. The selectivity of an assay refers to the extent to which the method detects the target analyte in the presence of interferents, such as enzyme inhibitors or degradants. The inclusion of IC-RNA in the triplex RT-PCR allowed exclusion of such samples reducing the potential for false-negative results.

### Detection in experimental and field samples

A total of 294 serum, EDTA and tissue samples were collected from 202 experimental and field animals infected with either CSFV or ASFV or deemed as negative samples as detailed in section 2.2. Nucleic acid was extracted and analysed with both reference method PCR tests and the triplex RT-PCR. The 228 samples analysed for CSFV detection with the triplex RT-PCR resulted in 191 concordant, 33 inconclusive and 4 false-positive results when compared with the CSFV RT-nPCR-TaqMan assay. The 164 samples analysed for ASFV with the triplex RT-PCR produced 134 concordant and 23 inconclusive results compared to the ASFV-PCR. Seven samples were detected as ASFV positive with the triplex RT-PCR which were not detected by the ASFV-PCR (Table 3). The 7 ASFV and 4 CSFV false positive results were either experimentally infected animals with clinical signs or samples that had tested positive in other tests and indicate that the triplex RT-PCR has a greater sensitivity than the 2 reference methods.

**Table 3:** Comparison of triplex RT-PCR with reference RT-PCR and PCR assays for detection of CSFV and ASFV in experimental and field samples.

		TRIPLEX RT-PCR		
		CSFV Positive	CSFV Negative	Inconclusive
**CSFV RT-nPCR-TaqMan**	**CSFV Positive**	102	0	15
	**CSFV Negative**	4	89	18
		**ASFV Positive**	**ASFV Negative**	**Inconclusive**
**ASFV-PCR**	**ASFV Positive**	47	0	5
	**ASFV Negative**	7	87	18

### Diagnostic sensitivity and specificity

The diagnostic sensitivity and specificity of the triplex RT-PCR was determined using reference samples which had been defined as positive or negative using the CSFV RT-nPCR-TaqMan and the ASFV-PCR assays. Multiple samples obtained serially from the same animal are not acceptable for establishing estimates of diagnostic sensitivity or specificity [30]. Therefore, nucleic acid extracted from a total of 146 EDTA blood, 7 serum and 49 tissue homogenates from 202 individual animals, was used for calculation of diagnostic sensitivity and specificity.

Of the 166 animals tested for the presence of CSFV using the RT-nPCR-TaqMan, 137 of these samples resulted in concordant results with the triplex RT-PCR, whilst 29 samples produced an “inconclusive” result in the triplex RT-PCR (Table 4). The 122 animals analysed with the ASFV-PCR resulted in 106 concordant results with the triplex RT-PCR, 14 inconclusive results and 2 false positive results (Table 4). As samples that produce an inconclusive result would be investigated further, or additional samples requested prior to diagnosis, these samples were excluded from analysis of diagnostic sensitivity and specificity.

**Table 4:** Diagnostic sensitivity and specificity estimates of triplex RT-PCR compared to reference RT-PCR and PCR assays for CSFV and ASFV.

		TRIPLEX RT-PCR		
		CSFV Positive	CSFV Negative	Inconclusive
**CSFV RT-nPCR-Taqman**	**CSFV Positive**	62	0	15
	**CSFV Negative**	0	75	14
		**ASFV Positive**	**ASFV Negative**	**Inconclusive**
**ASFV-PCR**	**ASFV Positive**	32	0	1
	**ASFV Negative**	2	74	13

For both CSFV and ASFV detection, the triplex RT-PCR had a 100% diagnostic sensitivity. Diagnostic specificity estimates were 100% for CSFV detection and 97.3% for ASFV detection.

## Discussion

Transboundary diseases, such as CSF and ASF, have significant economic impact and high mortality rates. This, coupled with comparable clinical presentations for both diseases, justifies the requirement for a sensitive and accurate differential diagnosis to detect and control subsequent spread of either disease. Current diagnosis of both diseases is based on virus or viral nucleic acid isolation from whole blood or tissue samples and/or antigen or antibody detection from serum samples [31], [32]. The recent outbreaks of ASFV close to European borders highlights the possibility of incursion into the EU and, in 2012, this was confirmed when backyard pigs in Ukraine exhibiting non-specific clinical signs were diagnosed with ASFV [3]. The spread of ASFV close to, and into, regions where CSFV is endemic in wild boar populations [33] and the emergence of CSFV in countries such as South Africa where ASFV is endemic [34], further substantiates the need for a simple differential diagnostic test that can detect both diseases without additional effort.

Various gel-based PCR and QPCR or RT-PCR assays have been described for the detection of ASFV [19]–[21], [35] or CSFV [12]–[14], [36]–[38]. Whilst both conventional and real-time multiplex PCR assays are published for the detection of ASFV [10], [11], [19], [39] or CSFV, [11], [26], [40], [41] alongside detection of other viruses and/or internal controls, to date simultaneous detection of CSFV and/or ASFV in a one-tube, real time RT-PCR format has not been reported.

Development of multiplex RT-PCR assays is not a simple procedure and provides a greater challenge than designing singleplex assays. The technique often requires extensive optimisation as primer-dimers and non-specific amplicons may interfere with amplification of the desired targets. Additionally, it is important that potential detection of two or more amplicons does not result in an impaired or preferential amplification of target nucleic acids [42]. It is also imperative that the dynamic range of the assay is broad enough to encompass most diagnostic samples.

This study details the optimisation and evaluation of a novel, multiplex real time RT-PCR assay for the differential diagnosis of CSFV and/or ASFV alongside an exogenous internal control using a single extraction methodology suitable for nucleic acid isolation from both the RNA CSFV and DNA ASFV. By combining a modified version of a protocol for CSFV detection alongside an exogenous internal control [26] with a novel real-time assay for ASFV detection we have developed a one-step, triplex real time RT-PCR assay for simultaneous differential detection of the two viruses.

Combining multiple primers and probes did not significantly affect the efficiency of the triplex RT-PCR in comparison to corresponding singleplex and duplex assays for CSFV and ASFV. The dynamic range of the new assay encompasses the copy numbers expected in diagnostic samples and experimental animals were detected as infected at equivalent time points post infection to reference PCRs. The triplex RT-PCR resulted in lower C_T_ values for detection of ASFV DNA compared to the ASFV-PCR, indicating an increased sensitivity. The new ASFV primers amplify a much smaller region of the ASFV p72 gene than the primers used in the ASFV-PCR, which will improve the amplification efficiency and analytical sensitivity of the assay. The triplex assay, like other recently described singleplex ASFV PCRs [21], [39] therefore represents an improvement on the ASFV-PCR for highly sensitive ASFV detection.

The specificity of the primers and probes used in the triplex RT-PCR for detection of CSFV have been extensively analysed against a large number of different pestiviruses and diagnostic samples [12], [43]. As expected the slight modification of the reaction mix and addition of primers to detect ASFV did not affect this specificity.

The new assay detected representatives of 8 of the currently described 22 ASFV genotypes. Blast searches of the new ASFV primers confirm they have 100% identity with all ASFV p72 DNA sequences present in the current NCBI nucleotide collection, apart from 3 strains which have 2 nucleotide divergences to the ASFVp72IVI_L primer. One of these strains, Moz64, was detected by the new assay indicating that the assay will detect all currently characterised ASFV genotypes.

Diagnostic sensitivity estimates for the triplex RT-PCR using positive and negative samples from animals infected with CSFV or ASFV either in the field or experimentally, as well as samples taken from animals from the UK which is free of both CSFV and ASFV, resulted in 100% diagnostic sensitivity for detection of both CSFV and ASFV. Diagnostic specificity estimates demonstrated 100% and 97.3% specificity for CSFV and ASFV, respectively. Diagnostic specificity evaluates the reliability of the test to identify negative results compared to a reference method assay. A specificity of less than 100% can be obtained if the new test is more sensitive than the reference method test. The increased analytical sensitivity of the triplex RT-PCR assay for ASFV resulted in detection of a number of samples with low virus loads which were not detected with the ASFV-PCR. This is the reason a diagnostic specificity of less than 100% was obtained for the triplex RT-PCR for ASFV detection.

To identify false negative results due to PCR inhibition or ineffective nucleic acid extraction, an exogenous internal control was incorporated. The internal control is added to samples prior to extraction and acts as both a nucleic acid extraction and PCR amplification control. Samples containing high viral copy numbers of either CSFV or ASFV can result in an absence of IC-RNA amplification, however this does not impede the assay as the IC-RNA is included to detect “false-negative” results only. Absence of a C_T_ value for the internal control for a CSFV and ASFV negative sample indicates a problem and the result is therefore deemed as “inconclusive”. The amplification-limiting concentration of primers used, alongside the low final IC-RNA concentration, ensures inclusion of the IC-RNA does not interfere with the amplification of target viral nucleic acid.

From a total of 294 samples that were analysed during the optimisation of this assay, 41 samples resulted in “inconclusive” results, where C_T_ values for CSFV or ASFV indicate the samples were virus negative but IC-RNA probe-specific C_T_ values were ≤38. This percentage of “inconclusive” results was higher than was expected and has not been reported by others using the Hoffmann et al. (2005) CSFV duplex assay [44].

Inhibition of RT-PCR and PCR reactions is a recognised problem and can be caused by numerous substances, each with a different inhibitory mode of action [45]. The triplex RT-PCR assay described utilises nucleic acid extracted from a number of different sample types, including EDTA blood and homogenised tissue samples, using the QIAamp Viral RNA Mini kit (QIAGEN) which extracts both RNA and DNA. The current diagnostic sample provided for CSFV or ASFV PCR analysis is EDTA blood. Although the QIAamp Viral RNA mini kit has been used successfully for both ASFV and CSFV detection from EDTA blood [15], [19], EDTA blood is not a sample type recommended by the manufacturer. In addition to this, anticoagulants such as EDTA chelate Mg^2+^ within the RT-PCR mastermix which are an essential co-factor for DNA polymerases [46] and natural components within the blood, mainly heme [47] and leukocyte DNA [48], can inhibit the polymerase chain reaction. It is also possible that a residual carry-over of guanidine salts present in the lysis buffer could result in general reduction in amplification efficiency. Alternatively, the integrity of nucleic acid, including the naked IC-RNA is sensitive to degradation, for example due to ineffective inactivation of RNAses by the lysis buffer. Ineffective nucleic acid extraction can also occur with poor quality or clotted samples.

These factors could explain the higher than expected number of “inconclusive” results that have been seen during the optimisation of the triplex RT-PCR. Neither the CSFV RT-nPCR-TaqMan nor the ASFV ASFV-PCR assays include an internal control for monitoring extraction or amplification efficiency. Therefore, false-negatives due to PCR inhibition or inefficient nucleic acid extraction are not identified by these methods. Whilst an “inconclusive” result with the triplex RT-PCR means that a sample will need to be re-tested, this is more favourable than a false negative result due to PCR inhibition. As both viruses are cell associated EDTA blood is the sample used in the UK for CSFV and ASFV PCR diagnosis. However, both viruses can also be detected in serum by RT-PCR and PCR [10], [21], [44], [49]–[51]. Serum is routinely used for serological analysis of both viruses [31], [32] and use of this sample matrix for diagnostic PCRs may provide an alternative less prone to PCR inhibition than EDTA blood, and would also reduce the number of sample types required.

A high proportion of inconclusive results will impact on test turnaround times and is not ideal for the rapid reporting of results needed for effective disease control. Further improvements to the nucleic acid extraction method, such as use of methods that extract total nucleic acid without the use of chaotrophic denaturing agents such as guanidine thiocyanate, could reduce the percentage of inconclusive results produced.

In summary, we have demonstrated the use of a single nucleic acid extraction methodology alongside a one-step, real time RT-PCR for the differential diagnosis of CSFV and ASFV that includes an exogenous internal control for the detection of “false-negative” results. The assay is suitable for routine diagnosis of both porcine viruses and is able to detect CSFV and ASFV to 4.9 and 22 copies, respectively. There is no loss in diagnostic sensitivity compared to the CSFV RT-nPCR-TaqMan and ASFV PCR methods and no lack of specificity for detection of the two different target viral nucleic acids. The recent occurrence of both CSFV and ASFV in ever closer geographical areas highlights the necessity for a technique to rapidly and differentially detect these two diseases.
